# HIV Induces TRAIL Sensitivity in Hepatocytes

**DOI:** 10.1371/journal.pone.0004623

**Published:** 2009-02-27

**Authors:** Challagundla K. Babu, Kanitta Suwansrinon, Gary D. Bren, Andrew D. Badley, Stacey A. Rizza

**Affiliations:** 1 Division of Infectious Diseases, Mayo Clinic, Rochester, Minnesota, United States of America; 2 Program in Translational Immunovirology and Biodefense, Mayo Clinic, Rochester, Minnesota, United States of America; New York University School of Medicine, United States of America

## Abstract

**Background:**

HIV infected patients have an increased susceptibility to liver disease due to Hepatitis B Virus (HBV), Hepatitis C Virus (HCV), alcoholic, and non-alcoholic steatohepatitis. Clinically, this results in limited options for antiretroviral therapy and accelerated rates of liver disease, causing liver disease to be the second leading cause of death for HIV infected patients. The mechanisms causing this propensity for liver dysfunction during HIV remains unknown.

**Methodology/Principal Findings:**

We demonstrate that HIV and/or the HIV glycoprotein gp120 ligation of CXCR4 on hepatocytes selectively up-regulates TRAIL R2 expression and confers an acquired sensitivity to TRAIL mediated apoptosis which is mediated by JNK II, but not p38 nor G-proteins.

**Conclusions/Significance:**

These findings suggest that HIV infection renders hepatocytes more susceptible to liver injury during disease states associated with enhanced TRAIL production such as HBV, HCV, or steatohepatitis.

## Introduction

Liver disease has become the second most common cause of death of HIV infected patients [1], and the risk of liver related death increases with progressive HIV associated immunodeficiency [2]. This observation allows for the possibility that the pathogenic mechanisms which promote T cell depletion may also promote liver injury.

In HIV infected patients, liver disease occurs most commonly from Hepatitis C Virus (HCV) co-infection, active Hepatitis B Virus (HBV) infection, non-alcoholic steatohepatitis (NASH) [3], as well as alcohol associated steatohepatitis.

Both HIV associated T cell depletion as well as liver injury are regulated, at least in part, by disordered apoptosis. In particular, in both circumstances, the apoptosis inducing ligand TRAIL (TNF related apoptosis inducing ligand) as well as its cognate receptors have been implicated. TRAIL exerts apoptotic effects by binding to and signaling either TRAIL Receptor 1 and/or TRAIL Receptor 2 expressed on the surface of target cells. Each of HIV, HBV, and HCV alter the sensitivity of cells they infect by modulating expression of these receptors [4]–[6]. Consequently, diseases caused by each of these viruses are thought to be mediated, in part, by altered TRAIL: TRAIL receptor interactions. Moreover, in animal models, interrupting TRAIL: TRAIL receptor signaling ameliorates CD4 T cell depletion of HIV infected PBL-NOD-SCID mice [7], acute liver injury following HBV transfection [8] and steatohepatitis following acute viral infection and alcohol use [9].

It remains unknown how the HIV infection accelerates the disease course of HCV infection compared to mono-HCV infection. While it has been suggested that the immunodeficiency associated with HIV infection may be the reason HCV progresses more rapidly, two lines of evidence suggest this may not be the case: first, HCV progression is faster in HIV infected patients with normal immune function [10], [11] and second, the incidence and progression of other chronic infections, for example, *Helicobacter pylori* is no faster in HIV infected individuals [12].

Based upon the combined observations that 1) HCV progression is faster in HIV co-infection; 2) this effect is not necessarily solely due to immunodeficiency; and 3) TRAIL dysregulation characterizes both HIV disease and HCV liver injury, we hypothesized that the ability of HIV to modulate TRAIL signaling in T cells might also occur in hepatocytes. We therefore tested whether HIV particles or HIV proteins altered either TRAIL receptor expression or TRAIL sensitivity of the human hepatocyte cells, Huh7.

## Materials and Methods

### Chemicals and Reagents

Dulbecco's modification of Eagle's medium (DMEM) (Mediatech Inc., Manassas, VA). Fetal bovine serum (FBS) (Atlanta Biologicals Inc., Lawrenceville, GA). JNK II inhibitor, Pertusis toxin (PT), SB 203580 (Calbiochem and, San Diego, CA). Recombinant superkiller TRAIL(skTRAIL) (Alexis Biochemicals, San Diego, CA). Recombinant HIV-1 IIIB envelope glycoprotein 120 (gp120) and recombinant human T- cell receptor sCD4 (Immuno Diagnostics Inc., Woburn, MA). Bovine serum albumin (BSA) (Sigma Chemical Co, St. Louis, MO), anti-actin and anti-tubulin antibodies (Molecular Probes, Carlsbad, CA). AMD3100 (NIH AIDS Research and Reference Reagent Program, Germantown, MD). Antibodies to TRAIL R1, R2, R3, R4 for flow cytometry (BD Bioscience, San Diego, CA) and TRAIL R2 for Western blot (Capralogics Inc., Hardwick, MA), active caspase-3 (BD Biosciences). The MTS, CytoTox96 non-radioactive cytotoxicity assay kit (Promega, Madison, WI).

### Cell Viability

The human hepatocyte cell line Huh7 (a kind gift from Dr. G. Gores, Mayo Clinic, Rochester, MN) were cultured at 37°C DMEM with 10% FBS and 10,000 µg/ml penicillin/streptomycin, and 200 mM glutamine. The Huh7 cells were plated at a density of 0.1×10^5^ cells/well in 96-well plates in a final media volume of 200 µL. Cells were pre-incubated with either AMD3100 (10 µM), Pertussis toxin (1 µg/µl), JNK II inhibitor (400 nM), or SB203580 (8 µM), a p38 inhibitor for one hour at 37 °C. Then, 5 µg/mL HIV glycoprotein 120 (HIV gp120) IIIB or purified X4 HIV IIIb was added for six hours followed by 100 ng/mL of skTRAIL for twelve hours. Cell viability was assessed by using 20 µL of the methanethiosulfonate reagent (MTS) by measuring absorption at 490 nm using spectrophotometric plate reader (EL800, Bio-tek instruments Inc., Winooski, VT). The mean viability was calculated and the results were expressed as percentage (%) of MTS reduction.

### Western Blots

Huh7 cells (4×10^5^) were treated with 10 µg gp120 for 0–3 hours and lysed in lysis buffer (20 mM Tris, pH 7.5, 150 mM NaCl, 2 mM EDTA, 0.1% Triton-X100, 2 µg/ml aprotinin, 5 µg/ml leupeptin, 1 µM PMSF and 1 µM Saponin) on ice. 50 µg of protein was resolved on a 15% sodium dodecyl sulfate-polyacrylamide (SDS-PAGE) gel and electrotransferred to polyvinylidene fluoride (PVDF) membranes (Millipore Corporation, Bedford, MA). Membranes were blocked with 5% nonfat milk in TBST [Tris base (10 mM), NaCl (150 mM), Tween (0.1%), pH 7.5] for two hours at room temperature and probed with goat anti TRAIL R2 (1∶1000) antibodies overnight at 4°C. Actin (Sigma, St. Louis, MO) was used as a loading control and intensity was measured using EL Logic 2200 imaging software system.

### Flow Cytometry

Huh7 cells (4×10^5^) were harvested, washed in cold PBS, and fixed in 2% paraformaldehyde overnight at 4°C. Cells were permeabilized with 0.1% NP-40 for 15 minutes (active caspase-3) on ice, blocked with 2% BSA/PBS and stained with phycoerythrin (PE) conjugated anti human anti TRAIL R1, R2, R3, R4 mAb (1∶40) or rabbit polyclonal anti-active caspase-3 antibodies (1∶40) or isotype control (Abcam Inc., Cambridge, MA) for one hour on ice, and analyzed by flow cytometry (FACScan flow cytometer, BD Biosciences, San Diego, CA).

### Statistical Analysis

All results were expressed as the mean±standard deviation (SD) and repeated at least three times as indicated in the figures. Statistical comparisons were made by student's *t* test employed to calculate the significance between the paired observations. A *p* value of <0.05 was used as the level of significance [13].

## Results

### HIV induces hepatocytes to become TRAIL sensitive

**Figure 1:**
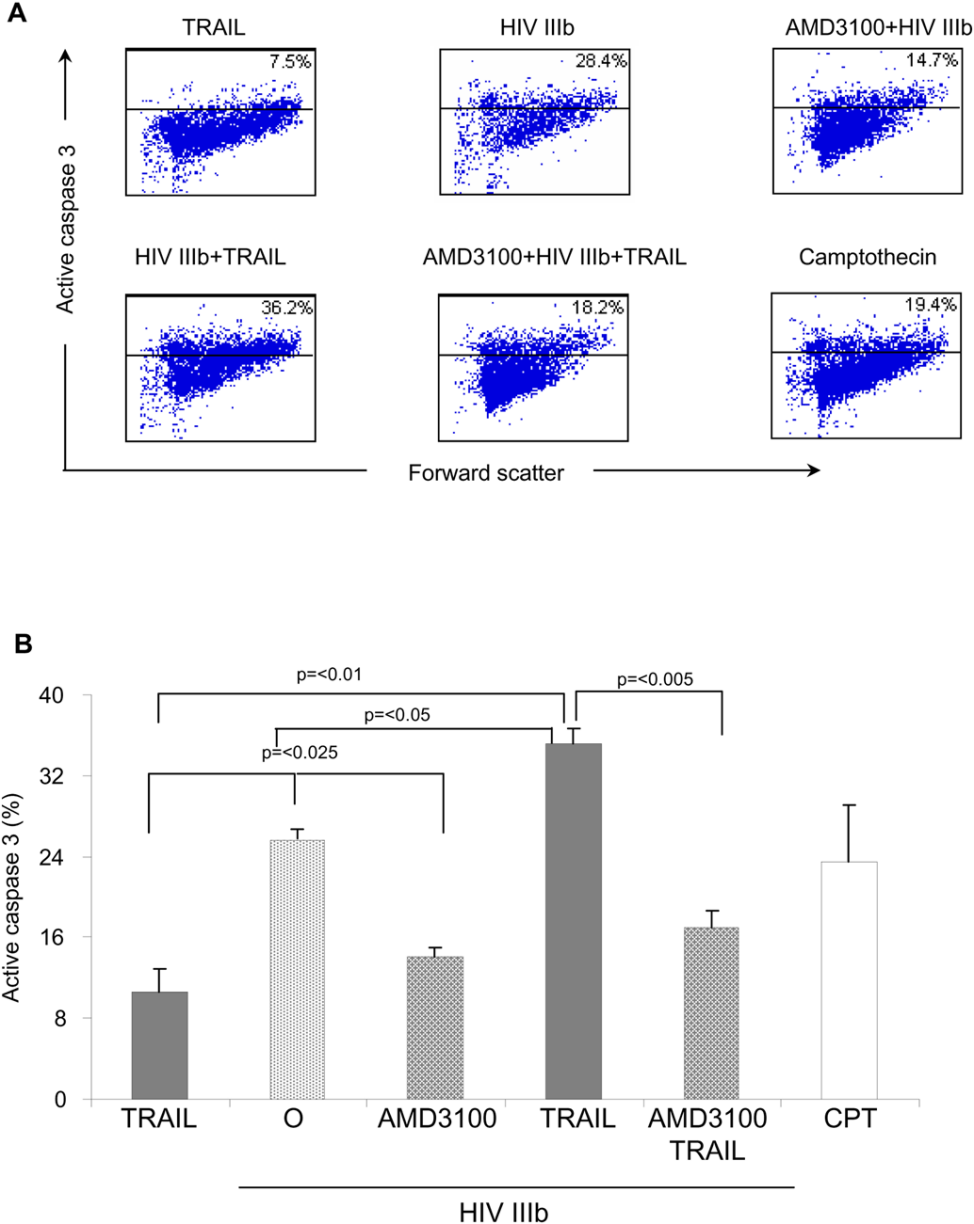
HIV IIIb makes Huh7 cells sensitive to TRAIL mediated apoptosis. Huh7 cells were pre-incubated or not with AMD3100 for one hour followed by treatment with purified HIV IIIb or mock virus for six hours. Selective points were then treated with skTRAIL for twelve hours. Cells were then fixed, permeabilized, and stained with fluorescently labeled anti-active caspase-3 antibodies and analyzed by flow cytometry. Camptothesin (10 µM) was used as positive control for caspase-3 activation. (A) A single representative dot plot for each group is given. In each plot, the percentage of cells positive for active caspase-3 is shown in the upper right quadrant. (B) Values are expressed as the mean±SD of three independent experiments.

In order to determine whether HIV alters the regulation of TRAIL sensitivity in hepatocytes, concentrated purified whole live HIV IIIb was used as a clinically relevant source of HIV gp120. Since Huh7 cells express the HIV receptor CXCR4, cells were pre-incubated or not with the CXCR4 receptor decoy AMD3100 and then treated with HIV IIIb. After six hours, skTRAIL was added, and the following day the cells were fixed, permeabilized, stained with antibodies that recognize the active form of caspase-3, and analyzed by flow cytometry. Untreated hepatocytes have spontaneous levels of apoptosis of <5%, while the addition of TRAIL increased apoptosis only minimally, confirming the relative resistance of these cells to TRAIL. In addition, and consistent with prior observations [14], [15], treatment with HIV IIIb causes 25% of human hepatocytes to directly undergo apoptosis. In contrast, treatment with HIV IIIb followed by TRAIL causes 34% apoptosis (p<0.05), arguing that HIV sensitizes cells to the pro-apoptotic effects of TRAIL. AMD3100 pre-treatment blocked both the direct HIV mediated death as well as the TRAIL induced death (p<.005), demonstrating that HIV IIIb mediated TRAIL sensitivity in human hepatocytes occurs via HIV gp120 binding to the CXCR4 co-receptor (Figure 1). Taken together, this data suggests that HIV gp120 binds to CXCR4 on the surface of a human hepatocyte, and signals the cell to become sensitive to TRAIL mediated apoptosis.

### HIV gp120 induces TRAIL R2 expression on the human hepatocyte cell line Huh7

**Figure 2:**
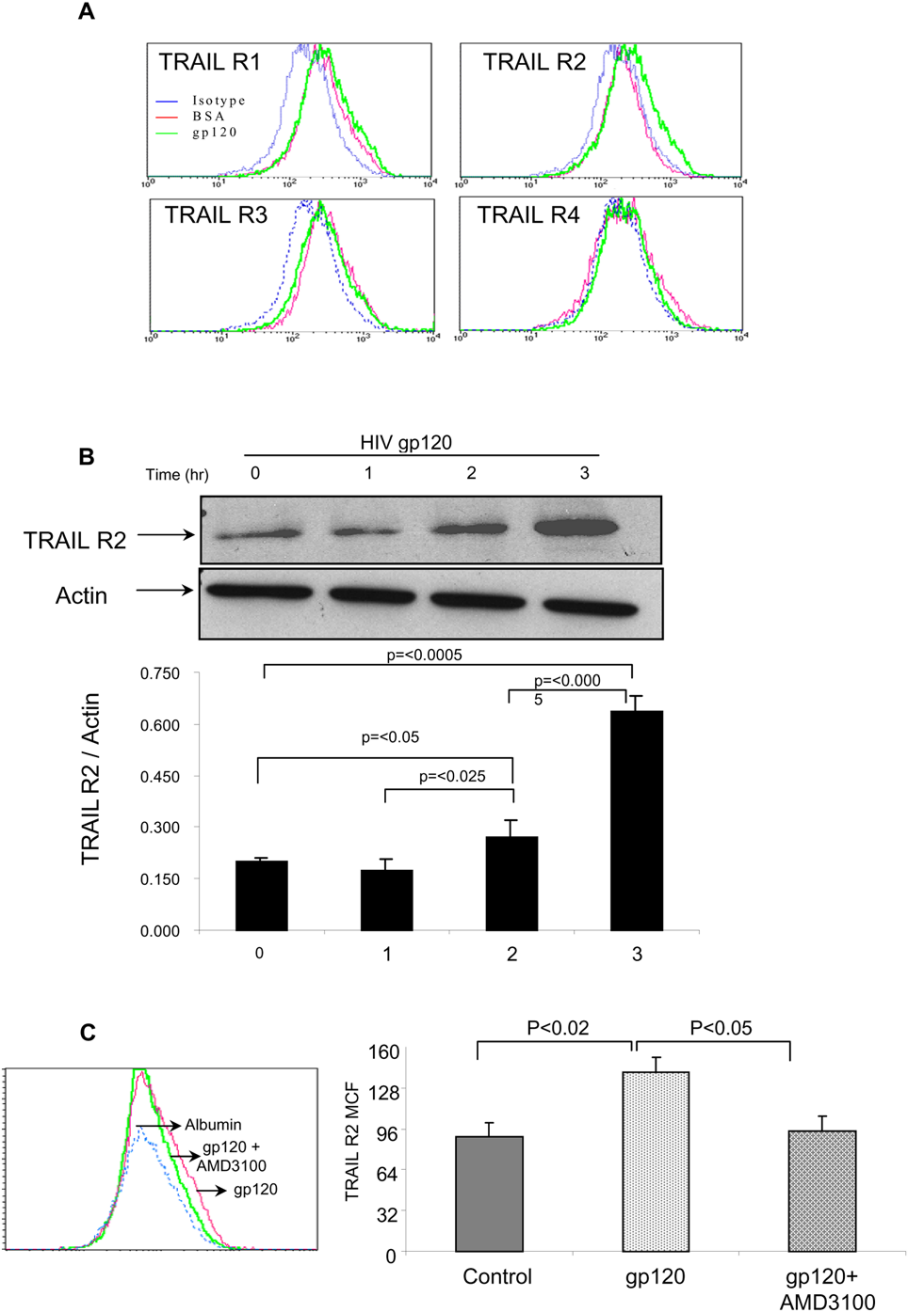
HIV gp120 up-regulates TRAIL R2 expression in Huh7 cells. (A) Huh7 cells were treated with HIV gp120 or control protein for six hours, stained with fluorescently conjugated antibodies specific for TRAIL receptors and analyzed by flow cytometry. BSA control protein is shown as a dotted line, and HIV gp120 is shown as a solid line. A single representative histogram from three independent experiments is given for each group. (B) Huh7 cells were treated with HIV gp120 for one, two, or three hours, lysed, and resolved on 15% SDS-PAGE, electrotransferred to PVDF membrane, and probed for TRAIL R2. Densitometer readings are presented as the relative intensity of TRAIL R2 normalized to actin. The data represents the mean±SD of three independent experiments. (C) Huh7 cells were pre-incubated or not with AMD3100 for one hour followed by treatment with HIV gp120 or BSA and analyzed for TRAIL R2. Data of mean channel fluorescence represents the mean±SD of three independent experiments.

Since human hepatocytes express the HIV chemokine co-receptor CXCR4 on the surface, [15] and since HIV gp120 treatment of immune cells [6], [16] increases TRAIL receptor expression, we questioned whether soluble HIV gp120, that is present during HIV infection, can signal through CXCR4 on the surface of human hepatocytes and also increase TRAIL receptor expression on the surface of the cell. When Huh7 cells, a human hepatocyte cell line, were incubated with control protein, TRAIL R1, R2, R3 or R4 expression was unchanged, yet pre-incubation of Huh7 cells with HIV gp120 resulted in a significant increase in TRAIL R2, but not R1, R3, nor R4 receptor expression as determined by flow cytometry (Figure 2A). Similarly, Western blot analysis confirms TRAIL R2 up-regulation after HIV gp120 incubation (Figure 2B). The effect of HIV gp120 on TRAIL R2 was abrogated by AMD3100, which binds CXCR4 and blocks HIV gp120 binding without signaling through the receptor (p<.005) [17]–[19] (Figure 2C). Consequently, the effect of HIV gp120 on TRAIL R2 is mediated by interaction with CXCR4.

### HIV gp120 renders Huh7 cells sensitive to TRAIL

**Figure 3:**
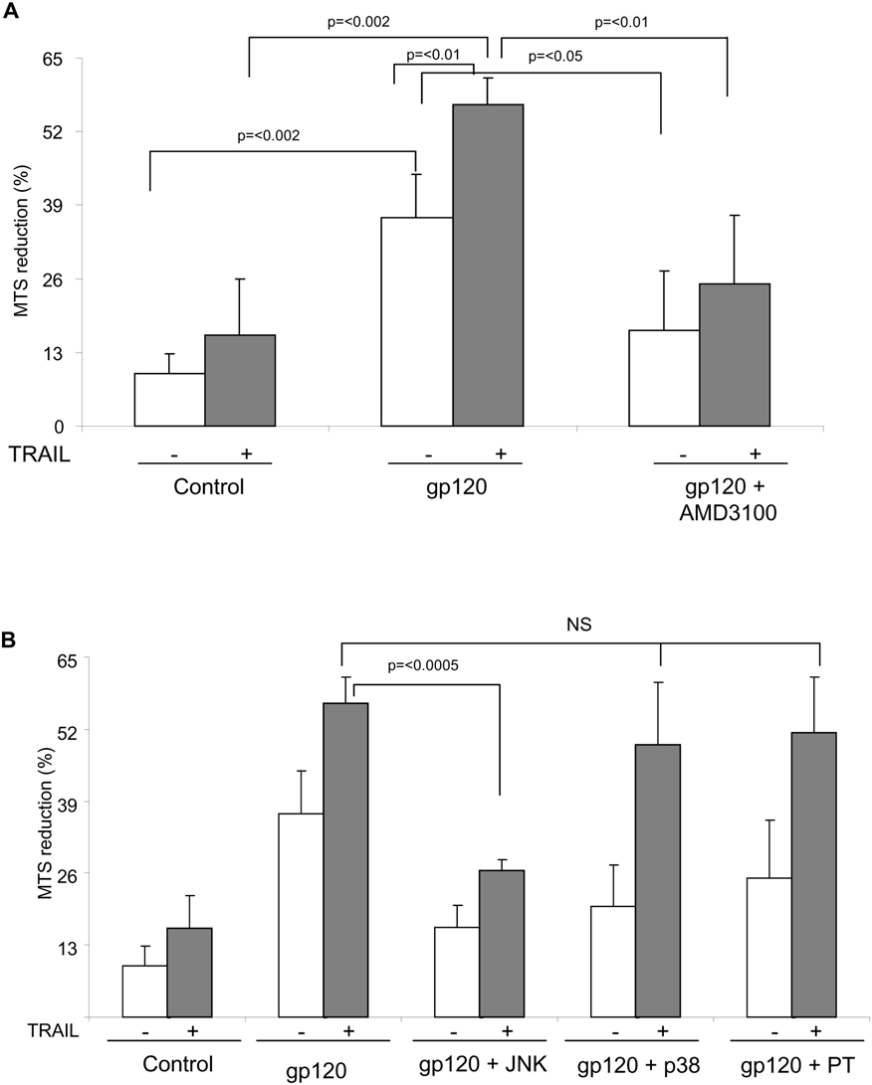
HIV gp120 induces TRAIL sensitivity in Huh7 cells in a JNK dependent manner. (A) Huh7 cells were pre-incubated or not with AMD3100 for one hour and then treated with HIV gp120 or BSA for six hours followed by treatment of skTRAIL. Cell viability was determined by reduction of MTS by live cells. (B) Huh7 cells were pre-incubated with JNK II inhibitor, p38 inhibitor or the G-protein inhibitor, Pertussis toxin, for one hour followed by treatment with HIV gp120 or control protein for six hours. Cell viability was determined by reduction of MTS in live cells. Values are expressed as the mean±SD of three independent experiments from duplicate wells.

Because individuals with HIV infection are more prone to liver disease than non-infected individuals, and given that HIV gp120 makes immune cells sensitive to bystander TRAIL mediated death, we sought to determine whether HIV gp120 also causes the hepatocytes to become sensitive to TRAIL mediated apoptosis. Human hepatocytes were incubated with HIV gp120 or control protein for six hours and then stimulated with TRAIL. HIV gp120 alone induces hepatocyte death (p<.002), as previously described [15]. TRAIL alone also caused minimal apoptosis. When TRAIL was added after HIV gp120 incubation, there was a significant loss of cell viability (p<.01) (Figure 3A), demonstrating that HIV gp120 signals hepatocytes to become sensitive to TRAIL mediated death. This acquired TRAIL sensitivity was blocked by AMD3100 pre-treatment of the cells (p<.01), confirming that the TRAIL sensitivity was mediated by gp120/CXCR4 interaction.

### HIV gp120 induced hepatocyte TRAIL sensitivity is mediated by JNK II kinase but not p38 kinase or the CXCR4 G-protein

HIV gp120 triggers a number of intracellular signals after binding CXCR4. Gp120/CXCR4 T cell death is associated with p38 phosphorylation and activation, [20] as well as JNK II kinase activity in a number of cell models [21], [22]. SDF1α, the natural ligand of CXCR4, induces chemotaxis through a G-protein dependent mechanism. We therefore investigated the role of JNK II, p38, and G-proteins on gp120 induced sensitivity to TRAIL. When Huh7 cells were treated with HIV gp120, direct hepatocyte death was abrogated by JNK II, p38, and G-protein inhibition, consistent with our prior observations [15] (Figure 3B). However, TRAIL sensitivity was blocked only when the cells were pre-incubated with JNK II kinase inhibitor (p<.0005), but neither p38 inhibitor, nor the CXCR4 associated G-protein inhibitor, nor Pertussis toxin (Figure 3B). In parallel experiments, Pertussis toxin blocked SDF1α induced T cell chemotaxis, and the p38 inhibitor blocked HIV gp120 induced p38 phosphorylation in T cells (data not shown), confirming the activity of these inhibitors. Consequently, JNK II kinase signaling is required for HIV gp120 conferring TRAIL sensitivity. Unlike direct HIV gp120/CXCR4 death signaling, acquired TRAIL sensitivity in hepatocytes does not require p38.

### HIV gp120 induced TRAIL R2 expression on hepatocytes is mediated by JNK II kinase but neither JNK I nor p38 kinase

**Figure 4:**
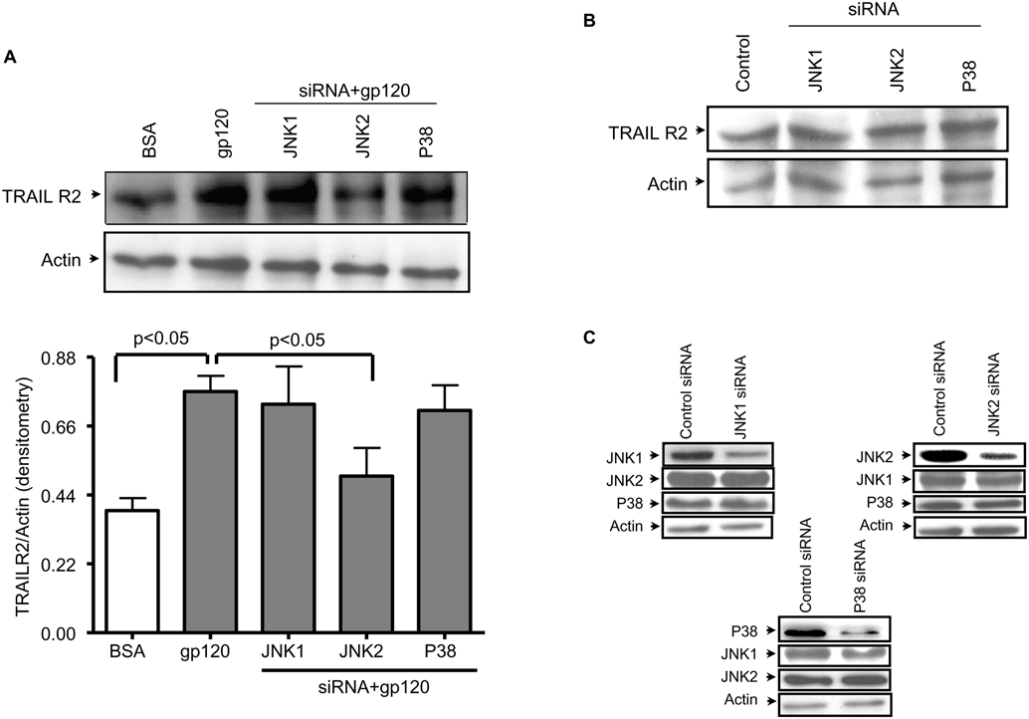
siRNA inhibition of JNK II inhibits gp120 induced TRAIL R2 upregulation. Huh7 cells were treated with either BSA or gp120 in the presence or absence siRNA constructs for JNK I, JNK II, and p38; and analyzed for TRAIL R2 content (A). The results presented below are pooled TRAIL R2 densitometry normalized to Actin. Parallel experiments demonstrate that siRNA for JNK I, JNK II, and p38 did not alter TRAIL R2 expression (B), and these siRNA constructs were specific for the proteins against which they were directed (C).

Given that HIV gp120 induced TRAIL sensitivity can be abrogated by pre-incubating hepatocytes with JNK II kinase inhibitors, we questioned whether the concomitant increase in TRAIL R2 expression on hepatocytes is also mediated by the JNK II kinase. Huh7 cells were transfected with siRNA for JNK I, JNK II, p38 kinase, or scramble control, followed by treatment with HIV gp120. After 18 hours of HIV gp120 treatment, the cells were lysed and blotted for TRAIL R2. Figure 4A demonstrates that JNK II inhibition by siRNA, but not p38 nor JNK I, decreases TRAIL R2 expression to the level of BSA control. Transfecting Huh7 cells with siRNA for JNK I, JNK II, or p38 did not intrinsically alter TRAIL R2 levels (Figure 4B). Furthermore, each siRNA inhibited the specific kinase without altering the levels of JNK I, JNK II, or p38 respectively (Figure 4C). Therefore, JNK II kinase is required for the HIV gp120 mediated increase in TRAIL R2 levels in Huh7 cells, as well as the conferred TRAIL sensitivity.

## Discussion

Liver disease is a frequent cause of morbidity and mortality in HIV infected individuals. Recently, the leading cause of death for hospitalized HIV patients in the United States became liver disease (MMWR) [23]. Multiple factors contribute to liver injury during the course of HIV infection. Many patients are co-infected with other viruses such as Hepatitis B, C, and D, as well as bacteria and mycobacteria. In addition, highly active antiretroviral therapy used during HIV infection is metabolized via the liver and can result in varying degrees of liver injury. Although the causes of HIV related liver disease are likely multifactorial, there are reports of liver injury resulting from HIV infection alone [24], [25]. Moreover, HIV RNA and the HIV antigen p24 have been isolated from liver samples, presumably from immune cells in HIV infected subjects, indicating that HIV virus and proteins are in close proximity to hepatocytes during HIV infection [24], [26].

During HIV infection, CD4^+^ T cells are depleted by multiple mechanisms; including HIV gp120 binding to the chemokine co-receptor CXCR4 and resulting in resting CD4^+^ T cell death [27], [28]. HIV gp120 kills non-immune cells as well, including neurons and hepatocytes [14], [15], [29], [30]. Gp120 ligation of CXCR4 can also alter the sensitivity of immune cells to TRAIL induced apoptosis by increasing TRAIL R2 expression on CD4^+^ T cells [16], or on neutrophils [6].

In the current report, we have extended the previous understanding of how HIV gp120 may contribute to hepatotoxicity. First, as previously reported, gp120 ligation of CXCR4 expressed on hepatocytes can cause hepatocyte death directly in a minority of cells [15]. Second, in those cells which survive, CXCR4 signaling results both in an up-regulation of TRAIL R2 expression and an acquired sensitivity to TRAIL induced death. This latter effect likely has implications for other hepatic disease states associated with TRAIL induced liver injury, and may explain why the course of such diseases is accelerated in HIV infected patients.

Since human hepatocyte cell lines as well as freshly isolated primary human hepatocytes express CXCR4 on the cell surface, we tested the hypothesis that HIV gp120 could bind to CXCR4 and up-regulate TRAIL R2 and consequently induce TRAIL sensitivity in human hepatocytes, thereby making the cells vulnerable to apoptosis in settings of high TRAIL expression (Figure 4). We show that consistent with prior reports, hepatocytes are not sensitive to TRAIL apoptosis [31]–[33]. Moreover, treatment with either HIV gp120 or whole virus induces an acquired sensitivity of hepatocytes to TRAIL mediated killing.

**Figure 5:**
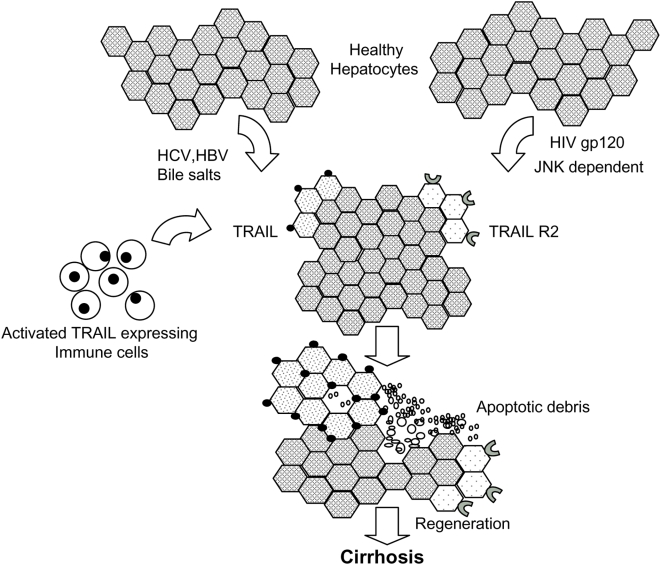
Role of TRAIL in HIV/HCV co-infection. Schematic model of synergy between HIV gp120/CXCR4-induced TRAIL sensitivity and other proapoptotic stimuli (e.g., HBV, HCV, Bile salts), causing hepatocyte death and consequent cirrhosis.

Taken together with reports that HBV infection [34], free fatty acids [35] and bile salts [36], or HCV [9], [37]can increase TRAIL expression, we propose a model (Figure 5) whereby HIV infection alone induces TRAIL sensitivity in human hepatocytes which acts synergistically with increased TRAIL expressed in other liver disease states, thereby accelerating liver disease. In addition, the enhanced TRAIL sensitivity of hepatocytes from HIV-infected patients would be favored to undergo apoptosis following exposure to TRAIL expressing immune cells. This is particularly relevant given that TRAIL levels are elevated during HIV infection [38], [39] and there is increased TRAIL expression on hepatocytes during infection with HCV [40], a common co-infection with HIV that results in accelerated liver disease.

HIV gp120 death and signaling can be mediated through several possible pathways. In immune cells, HIV gp120 binds CXCR4 and causes a G-protein independent death that signals via p38 [20]. In this hepatocyte model, HIV gp120 caused a small, direct death of hepatocytes, as we have previously described, that was p38 mediated, however, the HIV gp120/CXCR4 signaling that induces hepatocyte TRAIL sensitivity appears to signal differently. TRAIL sensitivity was independent of p38 and G-protein signaling but required JNK kinase, can activate the mitochondrial-dependent apoptosis pathway, and is involved in TRAIL R2 signaling [41]–[43].
